# Enhanced lubricin secretion by human synovial mesenchymal stem cells compared to adipose mesenchymal stem cells

**DOI:** 10.1186/s13287-025-04809-1

**Published:** 2025-11-26

**Authors:** Takuya Takakuwa, Kentaro Endo, Nobutake Ozeki, Yusuke Nakagawa, Hideyuki Koga, Makoto Tomita, Ichiro Sekiya

**Affiliations:** 1 Center for Stem Cell and Regenerative Medicine, Department of Applied Regenerative Medicine, Institute of Science Tokyo, 1-5-45 Yushima, Bunkyo-ku, Tokyo, 113-8510 Japan; 2 Department of Cartilage Regeneration Medicine, Graduate School of Medical and Dental Sciences, Institute of Science Tokyo, Tokyo, Japan; 3 Department of Joint Surgery and Sports Medicine, Graduate School of Medical and Dental Sciences, Institute of Science Tokyo, Tokyo, Japan; 4 School of Data Science, Graduate School of Data Science, Yokohama City University, Yokohama, Kanagawa Japan

**Keywords:** Adipose tissue, Knee, Lubricin, Mesenchymal stem cell, Osteoarthritis, Synovium

## Abstract

**Background:**

Mesenchymal stem cell (MSC) therapy has emerged as a promising treatment option for knee osteoarthritis. Adipose MSCs are commonly used due to their easy accessibility; however, synovial MSCs have demonstrated a superior capacity for cartilage matrix synthesis. The mechanism underlying this difference in therapeutic efficacy may involve lubricin, a crucial glycoprotein that maintains joint lubrication and protects cartilage. Notably, lubricin levels decrease during the progression of osteoarthritis. The aim of this study was to compare extracellular lubricin secretion by human synovial MSCs versus adipose MSCs. We also analyzed potential correlations between synovial MSC lubricin secretion and synovial inflammation.

**Methods:**

Tissues for MSC isolation were obtained from 16 human donors with osteoarthritis who underwent total knee arthroplasty. Synovium was collected from the suprapatellar pouch on the femoral side, whereas adipose tissue was harvested from the subcutaneous layer of the knee skin incision. The synovial and adipose MSCs from each donor were cultured for 48 h (six replicate wells per donor), and lubricin concentrations in culture supernatants were measured using ELISA. For 11 donors, lubricin concentrations were measured directly, whereas the concentrations were normalized to cell number for the other 5 donors. MSC identity was assessed by flow cytometry and trilineage differentiation assays. Correlations between synovial MSC lubricin secretion and clinical parameters, including age, CRP, WBC, numerical rating scale for knee pain, synovial redness, synovial hyperplasia, and Krenn’s synovitis score, were assessed.

**Results:**

Lubricin secretion was significantly greater from synovial MSCs than from adipose MSCs in 8 of the 11 directly analyzed MSC supernatants (p = 0.014 by Wilcoxon matched-pairs signed-rank test). However, the results from all 5 donors whose lubricin concentrations were normalized to cell number revealed a consistently higher lubricin secretion by synovial MSCs than by adipose MSCs. Every representative preparation of synovial and adipose MSCs fulfilled standard MSC identity criteria. No correlations were found between lubricin secretion and synovial inflammation or any clinical parameters.

**Conclusions:**

More lubricin was secreted from human synovial MSCs than from adipose MSCs in the majority of donors examined. These findings support the potential therapeutic advantages of using synovial MSCs for osteoarthritis treatment.

## Introduction

Osteoarthritis (OA) of the knee, the most common human joint disorder, is characterized by progressive cartilage degradation, synovitis, meniscal degeneration, and structural changes in the infrapatellar fat pad [1, 2]. Conventional treatments include nonsteroidal anti-inflammatory drugs, intra-articular hyaluronic acid injections, and exercise therapy, but these approaches often provide limited efficacy, resulting in an eventual requirement for arthroplasty. However, intra-articular injection of mesenchymal stem cells (MSCs) has now emerged as a promising alternative therapy with the potential to alleviate pain and improve clinical OA outcomes [3–5].

MSCs for clinical applications can be harvested from various tissues, including adipose and synovial tissues [6]. Adipose tissue has been a preferred source, by virtue of its availability from numerous easily accessed sites, such as subcutaneous, abdominal, and infrapatellar fat pads, and its ease of harvesting [7]. However, for knee OA applications, MSCs obtained from the knee synovium demonstrate higher capacities for chondrogenic differentiation, cartilage matrix synthesis, and effective cartilage repair [8–11], as demonstrated by successful cartilage repair in both animal models [12, 13] and human clinical studies [14]. In our previous study, human synovial and adipose MSCs were injected into the knees of rats with surgically induced osteoarthritis, revealing that the superior performance of synovial MSCs might be attributable to higher lubricin expression [15].

Lubricin is an intra-articular glycoprotein that is essential for joint lubrication and cartilage integrity. It plays a critical role in maintaining joint health [16–18]; however, its levels decrease as OA progresses [19–21]. MSCs are known to secrete lubricin [22, 23], but previous studies have mainly evaluated its expression, either at the gene level using PCR or by assessing the proportion of transplanted cells showing positive lubricin expression [15]. To date, no study has directly compared the actual amount of lubricin secreted extracellularly by synovial versus adipose MSCs, nor have possible relationships between MSC lubricin secretion and the clinical parameters associated with joint condition been explored. Therefore, the aims of the present study were to compare the extracellular secretion of lubricin between human synovial and adipose MSCs. We also analyzed the potential correlations between synovial MSC lubricin secretion and synovial inflammation.

## Materials and methods

### Ethics

This study was conducted in accordance with the criteria of the Declaration of Helsinki (2024) and was approved by the Medical Research Ethics Committee of Tokyo Medical and Dental University (now the Institute of Science Tokyo) (M2017-142). Written informed consent was obtained from all the study subjects.

### Patients

The inclusion criteria were patients with a diagnosis of knee osteoarthritis who underwent total knee arthroplasty (TKA) at our hospital between December 8, 2022, and April 10, 2025. The exclusion criteria were patients with infection, those with rheumatoid arthritis (RA), and those who underwent revision TKA.

### Clinical parameters

Preoperative assessments included blood tests (CRP and WBC) and the numerical rating scale (NRS) for knee pain. During surgery, the synovium at the femoral attachment of the suprapatellar pouch was evaluated based on macroscopic findings and classified into three grades according to redness (0: pale, 1: slightly red, 2: red) and hyperplasia (0: smooth, 1: irregular, 2: finger-like).

### Isolation of synovial and adipose MSCs

Synovium and adipose tissues were harvested from the knees of 16 donors with OA during TKA. The synovial membrane was collected from the suprapatellar pouch on the femoral side and adipose tissue from the subcutaneous layer under the skin incision [24]. The tissues were minced and digested in 3 mg/mL collagenase (Sigma-Aldrich, St Louis, MO, USA) solution for three hours at 37 °C.

**Fig. 1:**
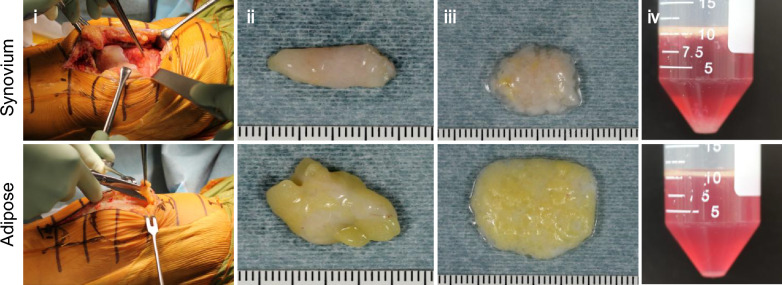
The process used for isolating human MSCs from synovial membrane and subcutaneous adipose tissue from harvest to enzymatic digestion. (i) Harvesting of synovium from the suprapatellar pouch and adipose tissue from the subcutaneous layer during knee arthroplasty. (ii) Harvested tissue. (iii) Tissue after mincing. (iv) Tissue in collagenase solution after enzymatic digestion

The digested material was filtered through a 70 μm cell strainer (Greiner Bio-One GmbH, Frickenhausen, Germany) (Fig. 1) to remove debris. The resulting nucleated cells were cultured in growth medium consisting of α-MEM (Thermo Fisher Scientific, Rockford, IL, USA), 1% antibiotic-antifungal agent (15240062, Thermo Fisher Scientific), and 10% fetal bovine serum (FBS) (Thermo Fisher Scientific) at 37 °C in 5% carbon dioxide (CO_2_). On day 14, the MSCs were detached with trypsin-ethylene diamine tetraacetic acid (EDTA) (Thermo Fisher Scientific) and stocked in 95% growth medium and 5% dimethyl sulfoxide (Fujifilm Wako Pure Chemicals, Osaka, Japan).

### Sample preparation

**Fig. 2:**
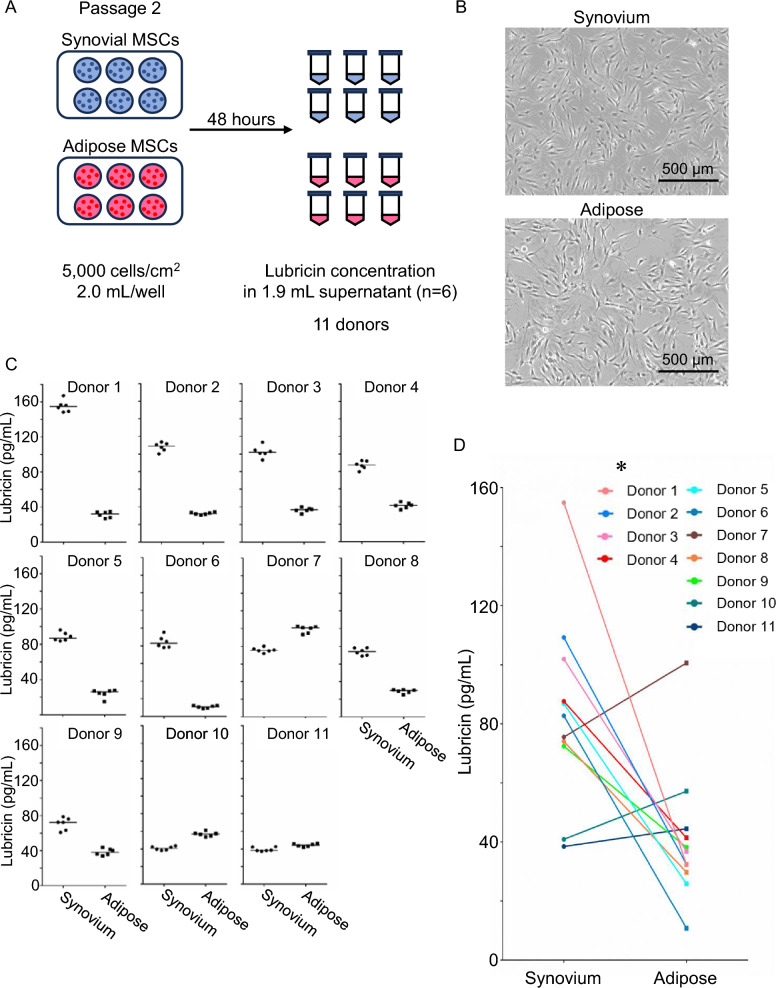
Lubricin secretion from synovial and adipose MSCs without normalization by cell number. **A** Experimental design. Passage 2 synovial MSCs and adipose MSCs from the same human donors were seeded at 5000 cells/cm^2^ in six-well plates (2.0 mL/well) and cultured for 48 h. Culture supernatants (1.9 mL/well) were collected, and lubricin concentrations were measured in six replicate wells per donor (n = 11 donors). **B** Representative morphology of synovial and adipose MSCs at 48 h after plating. Scale bars, 500 μm. **C** Lubricin concentrations in culture supernatants from each donor. Each dot represents an individual well (n = 6 per condition). Bars indicate median values. Donor numbers were assigned in descending order of lubricin concentrations in synovial MSCs. **D** Paired comparison of lubricin concentrations between synovial and adipose MSCs. Median values for each donor are connected by lines. *p = 0.014 by Wilcoxon matched-pairs signed-rank test (n = 11)

**Fig. 3:**
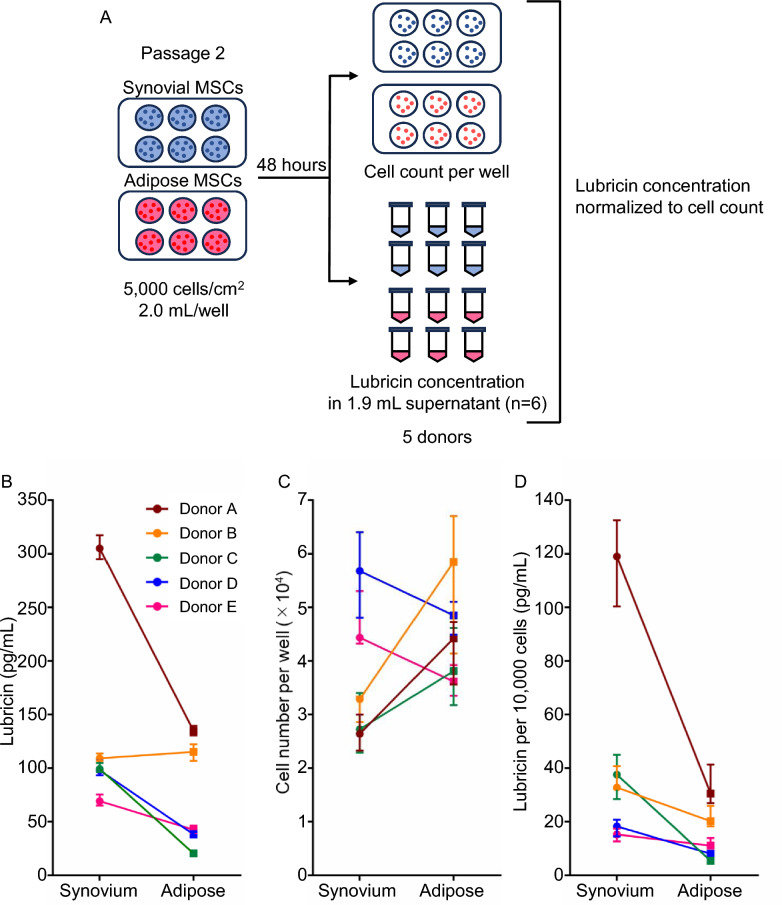
Lubricin secretion from synovial and adipose MSCs normalized to cell number. **A** Experimental design. Passage 2 synovial MSCs and adipose MSCs from the same human donors were seeded at 5000 cells/cm^2^ in six-well plates (2.0 mL/well) and cultured for 48 h. At the end of culture, cell numbers were determined in each well, and lubricin concentrations in 1.9 mL supernatants were measured in six replicate wells per donor. Lubricin concentrations were then normalized to cell numbers (n = 5 donors). **B** Lubricin concentrations in culture supernatants before normalization. Each line represents one donor, with values shown as median ± interquartile range from six replicate wells. **C** Cell numbers per well at 48 h for synovial and adipose MSCs. Each line represents one donor. **D** Lubricin secretion normalized to 10,000 cells. Each line, representing one donor, shows consistently higher lubricin secretion from synovial MSCs than from adipose MSCs

The MSCs stocked at passage 0 were thawed and cultured in growth medium at 500–1000 cells/cm^2^ in 145 cm^2^ dishes for passage 1. On day 7 of passage 1, the cells were detached and cultured at 5000 cells/cm^2^ in 9.6 cm^2^ wells for passage 2. Each MSC type was cultured in six wells containing 2.0 mL of growth medium per well. After 48 h, 1.9 mL of cell culture supernatant was collected from each well as a sample (Fig. 2A). For 5 out of the 16 donors, the number of cells in each well was measured at the time of supernatant collection using a Luna-FL^™^ automated cell counter (Logos Biosystems, Korea), and lubricin concentrations were normalized to the corresponding cell number (Fig. 3A).

### Quantification of lubricin concentration

The lubricin concentration in each sample was quantified using a commercially available enzyme-linked immunosorbent assay (ELISA) kit (Human Lubricin/PRG4, DuoSet ELISA, R&D Systems, Inc., USA) according to the manufacturer’s instructions. The assay was performed in 400 µL wells (96-well plates). Briefly, the wells were coated with 100 μL of capture antibody diluted in phosphate buffered saline (PBS) without carrier protein and incubated overnight at room temperature. After three washes with 400 µL of wash buffer, the plates were blocked with 300 μL of reagent diluent for 1 h at room temperature.

Following washing, 100 μL of standards or samples were added to duplicate wells and incubated for 2 h at room temperature. After washing, 100 μL of diluted detection antibodies were added and incubated for 2 h. Subsequently, 100 μL of diluted streptavidin–horseradish peroxidase (HRP) B solution was added and incubated in the dark for 20 min. After washing, 100 μL of substrate solution was added and incubated in the dark for 20 min, followed by the addition of 50 μL of stop solution.

The optical density was immediately measured at 450 nm and 560 nm using a microplate reader (Infinite M200; Tecan, Männedorf, Switzerland). The values were corrected by subtracting the 560 nm reading from the 450 nm reading. The average of duplicate wells was used for analysis. Standard curves were generated for each assay by plotting absorbance against concentration and fitting a curve through the points. The growth medium used in this study was confirmed by ELISA to be free of lubricin.

### Histology and immunofluorescence

Histological and immunofluorescence staining of synovium and subcutaneous adipose tissues was performed for 5 of the 16 donors. The tissues were fixed in 10% neutral-buffered formalin (Wako, Osaka, Japan), embedded in paraffin, and sectioned into 5 μm slices. The sections were deparaffinized with xylene and rehydrated through graded ethanol.

For histological analysis, hematoxylin and eosin (H&E) staining was performed, and images were acquired using a BZ-X800 microscope (Keyence, Osaka, Japan). Synovial inflammation was assessed using Krenn’s synovitis score [25].

For immunofluorescence analysis, antigen retrieval was performed in 10 mM Tris buffer containing 1 mM EDTA (pH 9.0) at 60 °C for 16 h. After blocking with Blocking One Histo (NACALAI TESQUE, Kyoto, Japan), the sections were incubated overnight at 4 °C with primary antibodies against lubricin (1:1000; MABT401, Sigma, MO, USA) and CD44 (1:200; 15,675–1-AP, Proteintech, Rosemont, IL, USA). After three washes with Tris-buffered saline containing 0.1% Tween-20 (TBS-T), the sections were incubated for 1 h at room temperature with Alexa Fluor 488– or Alexa Fluor 555–conjugated secondary antibodies (1:200; Abcam, Cambridge, UK). Nuclei were counterstained with DAPI (Dojindo, Kumamoto, Japan). Images were acquired using a BZ-X800 fluorescence microscope (Keyence, Osaka, Japan).

### Trilineage differentiation

Trilineage differentiation potential was confirmed in 3 out of 16 donors. For chondrogenesis, 2.5 × 10^5^ cells were placed into a tube with 500 μL of chondrogenic medium (n = 6), which was HG-DMEM (D6429, Sigma-Aldrich by Merck) supplemented with 50 μg/mL ascorbic acid 2-phosphate (A8960, Sigma-Aldrich), 40 μg/mL proline (P0380, Sigma-Aldrich), 1% antibiotic-antifungal agent, 100 nM dexamethasone (04718863, Wako), and 1% ITS + (354352, Corning), 10 ng/mL TGF-β3 (H2-1090, Proteintech), and 500 ng/mL BMP-2 (Medtronic). The tube was centrifuged at 580 × g for 10 min at room temperature, and the resulting pellets were incubated undisturbed at 37 °C in 5% CO₂ for 21 days. Cartilage pellets were collected, 10% formalin was added, and macro photography and wet weight measurements were performed.

For adipogenesis, 100 cells were plated in a 60 cm^2^ dish and cultured in growth medium consisting of α-MEM, 1% antibiotic-antifungal agent, and 10% FBS at 37 °C in 5% CO_2_ for 14 days. The growth medium was replaced with adipogenic medium, which was growth medium supplemented with 100 nM dexamethasone, 0.5 mM isobutylmethylxanthine (Sigma), and 100 µM indomethacin (Sigma), and the cells were cultured for an additional 21 days. Oil Red O staining was performed to confirm adipocyte differentiation.

For osteogenic differentiation, 100 cells were plated in a 60 cm^2^ dish and cultured in growth medium consisting of α-MEM, 1% antibiotic-antifungal agent, and 10% FBS at 37 °C in 5% CO_2_ for 14 days. The growth medium was replaced with osteogenic medium, which was growth medium supplemented with 1 nM dexamethasone, 50 µg/mL ascorbic acid 2-phosphate, and 10 mM β-glycerol phosphate (Sigma), and the cells were cultured for an additional 21 days. Alizarin red staining was performed to confirm osteoblast differentiation.

### Flow cytometry

Flow cytometry analysis was performed on passage 1 synovial MSCs and adipose MSCs obtained from the same donor. The cells were detached from dishes using TrypLE (Thermo Fisher Scientific, Rockford, IL, USA), collected, and washed twice with PBS. The cells were resuspended in FACS buffer (PBS containing 2% FBS and 5 mM EDTA) and incubated for 30 min at 4 °C in the dark with fluorochrome-conjugated monoclonal antibodies against CD73-FITC (561254, BD), CD90-PE (555596, BD), CD105-APC (562408, BD), CD44-APC-H7 (560532, BD), CD14-FITC (11–0149-42, Thermo), CD34-PE (550761, BD), CD19-APC (555415, BD), and CD45-APC-H7 (560178, BD). Corresponding isotype control antibodies were used to define background staining. After staining, the cells were washed with FACS buffer and filtered through a 35 µm cell strainer (Falcon, Corning, NY, USA) to remove clumps. Flow cytometry analysis was performed using a FACS Melody flow cytometer (BD, San Jose, CA, USA). Dead cells were identified and excluded using DAPI staining. Data were analyzed with FlowJo software (BD), and the proportion of antigen-positive cells was calculated after gating on viable cells.

### Statistical analysis

For the 11 donors without cell number normalization, lubricin secretion between synovial and adipose MSCs was analyzed with a significance level of 0.05, and the median values from each donor were compared using the Wilcoxon matched-pairs signed rank test.

For the additional five donors with cell number normalization, data were presented descriptively without statistical analysis due to the limited sample size.

The relationships between lubricin concentrations in culture medium from synovial MSCs and clinical parameters (age, CRP, WBC, NRS for knee pain, synovial redness, synovial hyperplasia, and Krenn’s synovitis score) were evaluated using Spearman’s correlation analysis.

All statistical analyses and graphs were performed using GraphPad Prism version 6.07 (GraphPad Software, Boston, MA, USA).

## Results

### Clinical parameters

The 16 donors consisted of 12 female and 4 male OA patients ranging in age from 58 to 87 years (median 75 years). The CRP level ranged from 0.03 to 0.84 mg/dL (median 0.18 mg/dL). WBC counts ranged from 3.5 to 7.8 × 10^3^ /μL (median 6.0 × 10^3^/μL). NRS for knee pain ranged in value from 2 to 10 (median 7). Synovial redness was pale in 3 donors, slightly red in 8, and red in 5. Synovial hyperplasia was smooth in 1 donor, irregular in 10 donors, and finger-like in 5 donors. Krenn’s synovitis score ranged from 0 to 2 (median 2) (Table 1).

**Table 1:** Demographic data, preoperative blood data, numerical rating scale, intraoperative synovial findings, and Krenn’s synovitis scores for 16 human donors who underwent total knee arthroplasty

Donor no	Gender	Age (years)	CRP (mg/dL)	WBC (× 10^3^/μL)	NRS for knee pain	Synovial redness	Synovial hyperplasia	Krenn’s synovitis score
1	Female	75	0.17	5.9		Pale	Irregular	
2	Female	69	0.84	7.8	7	Red	Finger-like	
3	Female	84	0.03	6.3	4	Slightly red	Irregular	
4	Female	87	0.04	6.2	7	Red	Irregular	
5	Female	77	0.10	5.1	4	Red	Finger-like	
6	Female	69	0.34	5.1	7	Slightly red	Irregular	
7	Female	81	0.21	5.9	9	Pale	Smooth	
8	Female	75	0.05	3.7	7	Red	Irregular	
9	Female	81	0.09	7.3	7	Slightly red	Irregular	
10	Male	75	0.10	4.2	3	Slightly red	Irregular	
11	Female	63	0.14	7.3	8	Slightly red	Finger-like	
A	Male	81	0.21	7.7	2	Red	Irregular	2
B	Male	74	0.19	6.0		Slightly red	Finger-like	2
C	Female	58	0.50	7.8	10	Slightly red	Irregular	0
D	Male	65	0.20	5.6	10	Slightly red	Finger-like	2
E	Female	75	0.69	3.5	8	Pale	Irregular	2

*CRP* serum C-reactive protein. *WBC* white blood cells. *NRS* numerical rating scale. For Donors 1–11, lubricin concentrations were measured without normalization to cell number, whereas for Donors A–E, the measurements were normalized to cell number

### Lubricin secretion without normalization by cell number

We first compared lubricin secretion between synovial MSCs and adipose MSCs without normalization by cell number (Fig. 2A). Passage 2 cells isolated from the same donors were cultured as six replicates under identical conditions for 48 h, and the lubricin concentrations in the culture supernatants were measured. Morphologically, both synovial and adipose MSCs exhibited a typical fibroblastic appearance (Fig. 2B). The variation in lubricin concentrations among the six samples from each donor appeared relatively small; however, lubricin secretion varied widely among donors (Fig. 2C). For synovial MSCs, the lubricin concentrations ranged from 37.4 to 167.2 pg/mL (median 80.3 pg/mL). For adipose MSCs, the lubricin concentrations ranged from 8.7 to 102.4 pg/mL (median 35.8 pg/mL). Lubricin secretion was greater from synovial MSCs than from adipose MSCs in 8 of 11 donors, with a statistically significant difference (p = 0.014, Wilcoxon matched-pairs signed rank test, n = 11) (Fig. 2D).

### Lubricin secretion with normalization by cell number

We next compared lubricin secretion from synovial and adipose MSCs after normalization to cell number (Fig. 3A). Before normalization, the lubricin concentrations in the culture supernatants were consistently higher for synovial MSCs than for adipose MSCs, with median values ranging from approximately 64.5 to 317.7 pg/mL (median 104.6 pg/mL) for synovial MSCs and from 18.3 to 150.7 pg/mL (median 42.8 pg/mL) for adipose MSCs (Fig. 3B). At the end of culture, the cell numbers per well were also higher for adipose MSCs (median 3.6–5.9 × 10^4^ cells) than for synovial MSCs (median 2.6–5.7 × 10^4^ cells) for all donors examined (Fig. 3C). To account for this difference, lubricin secretion was normalized to 10,000 cells. After cell number normalization, the secreted amounts of lubricin were still greater for the synovial MSCs, with values ranging from 12.0 to 132.7 pg/mL per 10,000 cells (median 32.2 pg/mL per 10,000 cells), than for the adipose MSCs, with values ranging from 3.5 to 43.3 pg/mL per 10,000 cells (median 11.1 pg/mL per 10,000 cells) (Fig. 3D). These results confirmed that the higher lubricin production by synovial MSCs did not simply reflect differences in cell proliferation.

### Histological characterization

**Fig. 4:**
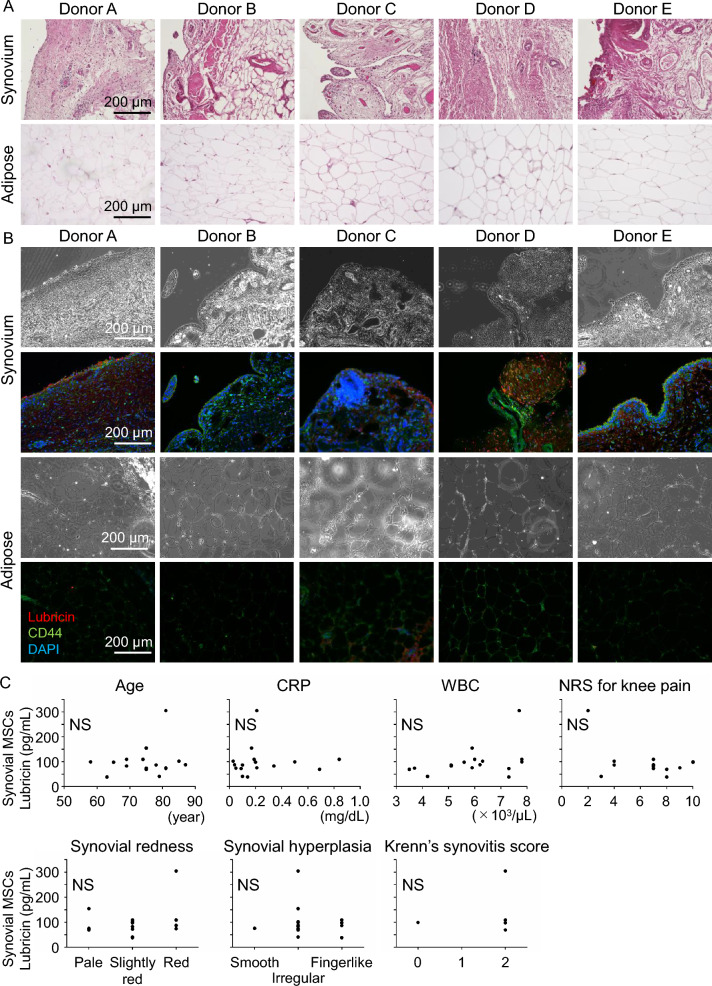
Histological characterization of human synovial and adipose tissues and correlation analysis between lubricin secretion and inflammatory parameters. **A** Histology of synovium and adipose tissues stained with hematoxylin and eosin (H&E). Scale bars, 200 μm. **B** Phase-contrast images (upper) and immunofluorescence staining (lower) of synovium and adipose tissues from the same donors. Lubricin (red), CD44 (green), and nuclei (DAPI, blue). Scale bars, 200 μm. **C** Correlations between lubricin concentrations in synovial MSC culture medium and clinical parameters, together with quantitative measures of inflammation. Systemic parameters included donor age, serum C-reactive protein (CRP), white blood cell count (WBC), and numeric rating scale (NRS) for knee pain. Local synovial parameters related to inflammation included synovial redness, synovial hyperplasia, and Krenn’s synovitis score. Synovial redness and hyperplasia were graded on a three-point scale during total knee arthroplasty, and Krenn’s synovitis score was assessed based on the H&E–stained histological sections shown above. Spearman’s correlation analysis revealed no significant associations between lubricin secretion and any of these parameters (*NS* not significant)

Histological examination of synovial tissue demonstrated variable features among donors, including differences in synovial lining thickness and stromal cellularity (Fig. 4A). In addition, numerous inflammatory cells were observed in the synovium, indicating inflammatory changes. In contrast, adipose tissue exhibited a uniform lobular architecture with adipocytes separated by thin fibrous septa, and no inflammatory cell infiltration was detected”.

Phase-contrast microscopy confirmed the tissue morphology, and immunofluorescence staining revealed strong lubricin expression in the synovial lining layer and surrounding stromal cells, co-localized with CD44 (Fig. 4B). In adipose tissue, lubricin staining was weak or nearly undetectable, while CD44 was present along the adipocyte membranes. These findings indicate that lubricin expression is characteristic of synovial tissue but not adipose tissue.

### Analysis between lubricin secretion and inflammatory parameters

We next investigated whether lubricin secretion from synovial MSCs was associated with systemic or local inflammatory parameters (Fig. 4C). Systemic parameters included donor age, serum CRP, WBC count, and NRS for knee pain. Local inflammatory parameters included intraoperative grading of synovial redness and synovial hyperplasia, as well as Krenn’s synovitis score assessed from H&E-stained sections. Spearman’s correlation analysis demonstrated no significant associations between lubricin secretion and any of these systemic or local parameters (all NS). Collectively, these results suggest that lubricin production by synovial MSCs is not influenced by patient background factors or the degree of synovial inflammation.

### Characterization of synovial and adipose MSCs

**Fig. 5:**
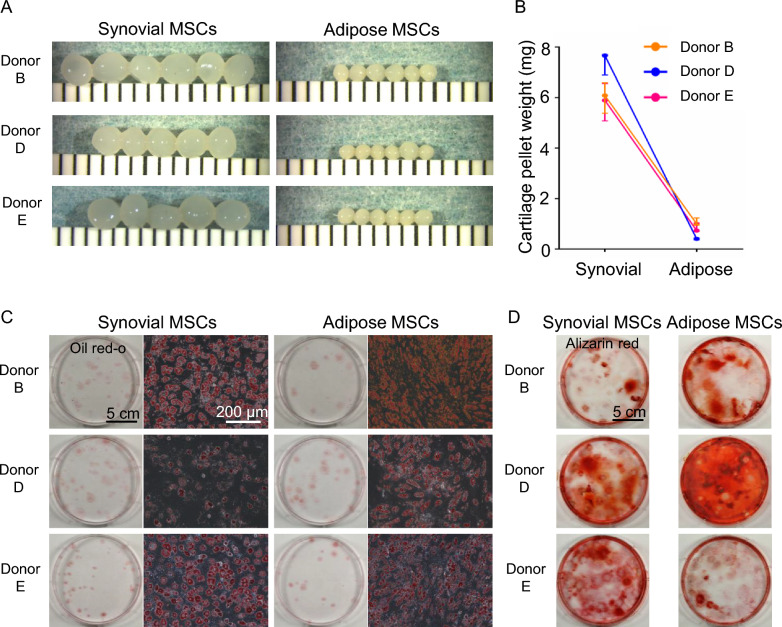
Differentiation potential of synovial and adipose MSCs. **A** Chondrogenesis. Macroscopic images of cartilage pellets formed by synovial MSCs and adipose MSCs from three donors. Five or six pellets per donor are shown. **B** Cartilage pellet weights. Each line represents one donor. Results are shown for three donors, and values represent the median of five or six pellets per donor with interquartile ranges. **C** Adipogenesis. Oil Red O staining of lipid droplets in synovial and adipose MSCs at the macroscopic (left) and microscopic (right) levels. **D** Osteogenesis. Alizarin Red staining of calcified matrix deposits in synovial and adipose MSCs

In chondrogenic differentiation, the cartilage pellets were larger and heavier when formed by synovial MSCs than by adipose MSCs in all three donors tested (Fig. 5A, B). The median pellet weight of synovial MSCs ranged from approximately 4.8 to 7.7 mg, whereas that of adipose MSCs ranged from 0.3 to 1.4 mg, with a clear separation across all donors. In adipogenic differentiation, Oil Red O staining demonstrated an accumulation of lipid droplets in both synovial and adipose MSCs, with no clear difference between the two cell types (Fig. 5C). In osteogenic differentiation, Alizarin Red staining revealed calcium deposition in both synovial and adipose MSCs, with no obvious difference in staining intensity (Fig. 5D).

**Fig. 6:**
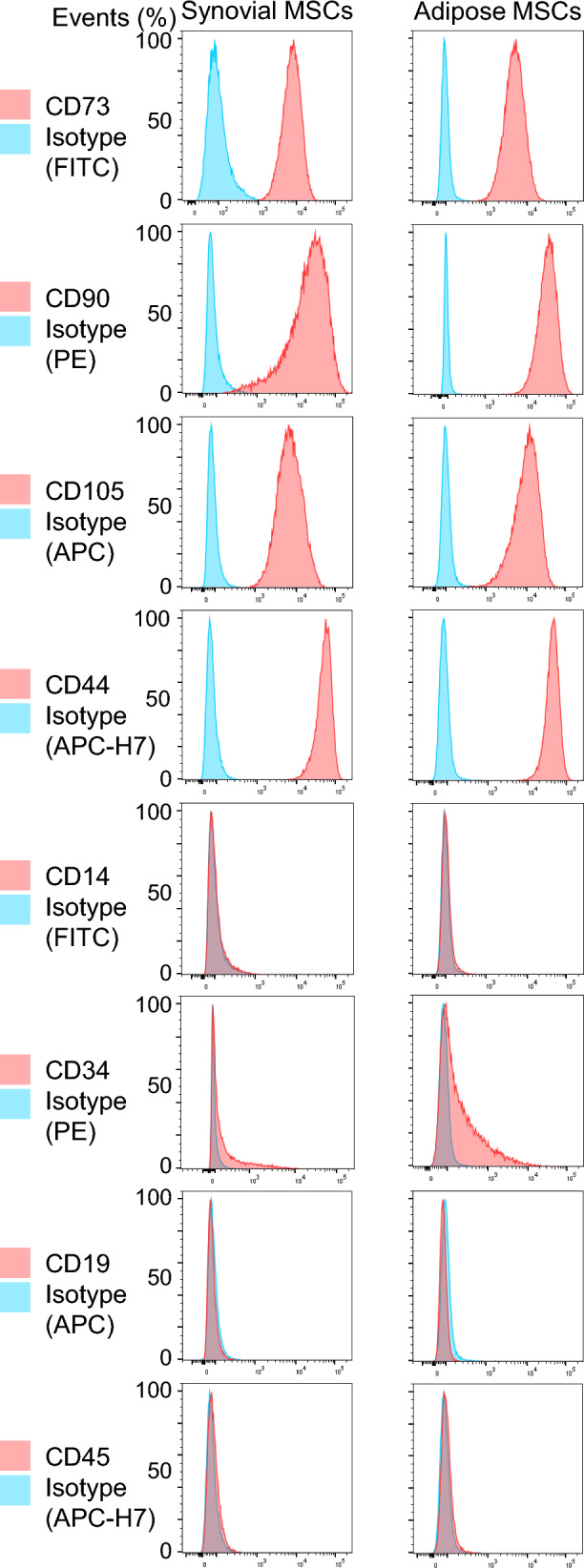
Flow cytometry analysis of surface marker expression in synovial and adipose MSCs. Representative histograms of surface antigen expression in synovial MSCs (left) and adipose MSCs (right)

Both synovial and adipose MSCs were strongly positive for the canonical MSC markers CD73, CD90, CD105, and CD44, while showing negative expression for the hematopoietic and endothelial lineage markers CD14, CD34, CD19, and CD45 (Fig. 6).

## Discussion

Significantly more lubricin was secreted by synovial MSCs than by adipose MSCs obtained from human donors. While considerable donor-to-donor variation was observed in lubricin secretion, the superior secretion by synovial MSCs was consistent across the majority of donors. We also found no correlations between the levels of lubricin secreted by synovial MSCs and the various clinical parameters of the patients.

Lubricin is considered the most essential substance in boundary lubrication [26], and its intra-articular concentration is known to decrease with OA progression [19–21]. In cell therapy for OA, synovial MSCs have demonstrated advantages over adipose MSCs derived from subcutaneous adipose tissue at the knee incision site by virtue of their superior capabilities for cartilage formation [8], apoptosis inhibition [27], and tissue repair, as well as their enhanced secretion of lubricin, as shown in the present study. Furthermore, the roles of lubricin extend beyond mere lubrication, as lubricin reportedly binds as an antagonist to toll-like receptors (TLRs), specifically TLR2 and TLR4, and inhibits the release of proinflammatory cytokines [28, 29]. These additional roles suggest that lubricin secreted by synovial MSCs may aid in suppressing OA progression through dual mechanisms involving physical lubrication and inflammation control.

Inflammation is a major pathological feature of OA [1], and inflammatory cytokines have been reported to promote synovial MSC proliferation. The significant increase in synovial MSC numbers in the presence of different inflammatory cytokines, such as tumor necrosis factor-α (TNF-α) and interleukin- 1β (IL-1β), suggests that this secretion represents an adaptive response to the inflammatory OA environment [30, 31]. We initially hypothesized that stronger inflammation would induce an increase in lubricin secretion by synovial MSCs as an anti-inflammatory response. However, in the present study, we found no correlation between gross synovial inflammation and lubricin secretion levels. This suggests that although the inflammatory environment promotes an increase in the number of synovial MSCs, thereby leading to greater total lubricin secretion, the secretory capacity of each individual cell remains unchanged.

Simple calculations, derived from the 11 samples without cell number normalization, show that 4.8 × 10^4^ synovial MSCs secrete 157.2 pg of lubricin over 48 h, suggesting that an intra-articular injection of 2.0 × 10⁷ MSCs would produce 65.5 ng of lubricin—or only 0.006% of the total lubricin in a healthy knee joint (1.1 mg, assuming a concentration of 364.4 μg/mL in 3 mL of synovial fluid) [19]. While this percentage appears small, several factors suggest that it has potential clinical significance because the lubricin levels are markedly reduced in OA knees [19–21], MSC-derived lubricin is likely secreted in a highly functional state, and MSCs proliferate in response to inflammatory cytokines [30, 31], potentially providing sustained lubricin production. Thus, the therapeutic impact of MSC-derived lubricin may be more significant than suggested by these initial calculations, particularly in the context of OA, where lubricin levels are depleted.

Adipose MSCs showed low lubricin expression, but previous studies have reported that their intra-articular injection induces therapeutic effects in OA knees through several mechanisms. For example, adipose MSCs reduce inflammation by secreting anti-inflammatory cytokines, such as IL-10, which suppress pro-inflammatory cytokines (e.g., IL-1β and TNF-α) in the joint [32, 33]. Adipose MSCs also exhibit immunomodulatory effects by shifting macrophages and T cells toward a repair-promoting phenotype, thereby improving the inflammatory environment [34, 35]. Adipose MSCs also secrete regenerative factors, such as transforming growth factor (TGF)-β, that promote the repair of cartilage and synovium [36]. These paracrine effects improve the joint environment, leading to pain reduction and functional improvement. Therefore, despite their low lubricin secretion, adipose MSCs function as an effective treatment for OA knees through alternative mechanisms.

Direct lubricin injection has been investigated in animal studies as a treatment for OA knees and has resulted in improved joint lubrication, reduced cartilage wear, and pain relief [37]. To our knowledge, no clinical studies are currently evaluating intra-articular lubricin injection in humans. (We have not performed intra-articular injection of adipose MSCs in humans; our experimental work has been limited to rats [15]. However, the instability of lubricin in vivo means that it may require frequent injections [38], and its high production cost remains an obstacle to its practical clinical implementation. By contrast, MSC therapy, which enables sustained lubricin production within the joint, may offer a more comprehensive treatment approach that overcomes the limitations of direct lubricin injection.

Of particular relevance to the findings of the present study is the substantial evolution of tissue harvesting methods for OA cell therapy since their inception. The advantage of using adipose MSCs lies in the relatively easy accessibility of adipose tissue from the abdomen. By contrast, procuring synovial tissue has traditionally required arthroscopic harvest from the knee—a relatively invasive procedure [39, 40]. However, the recent development of an ultrasound-guided technique for synovium tissue collection under local anesthesia has now rendered the invasiveness of synovium harvest comparable to that of adipose tissue collection [41].

An inflammatory phenotype of MSCs derived from adipose tissue of OA patients also remains a possibility, but previous studies have reported conflicting results for infrapatellar fat pad MSCs, likely a reflection of an influence of the inflamed joint environment [42]. To address this concern, we performed a histological evaluation of subcutaneous adipose tissue obtained at the knee incision site from five donors. Unlike synovial tissue, which exhibited inflammatory cell infiltration, the subcutaneous adipose tissue showed a uniform lobular structure without evidence of inflammatory findings in any donor. These observations suggest that—at least at the histological level—subcutaneous adipose tissue from OA patients does not display inflammatory changes.

We propose three limitations for this study. First, adipose MSCs were harvested from subcutaneous fat in the knee rather than from abdominal fat, which is the typical source for adipose MSCs. In this study, we chose knee subcutaneous fat at the incision site because it was available and easily obtained during TKA without additional invasiveness. However, this raises the possibility that differences in MSC characteristics between knee and abdominal subcutaneous fat might have influenced the properties of the MSCs [43, 44], and we cannot exclude the possibility that this variation in harvesting site affected our results. Second, our donors were limited to patients with end-stage knee OA who had undergone TKA. The results might differ if donors are in early-stage OA, as those patients are better candidates for cell therapy [45], suggesting that caution is needed when applying these findings to cell therapy for early-stage OA patients. Third, although significant differences were detected in some experiments, the number of donors was insufficient to confirm the overall findings, indicating the need for additional studies with larger sample sizes.

In conclusion, this study demonstrated that greater amounts of lubricin are produced by human synovial MSCs than by adipose MSCs, although individual donor variations were observed. Despite the inter-donor differences, the enhanced lubricin secretion by synovial MSCs remained a consistent finding. This characteristic of synovial MSCs, combined with their known cartilage repair capabilities, supports a potential therapeutic advantage for their use in OA treatment, although further investigation of the clinical implications is warranted.

## Data Availability

The datasets generated and analyzed during the current study are available from the corresponding author on reasonable request. Our study did not yield datasets suitable to include in online repositories.

## Abbreviations

BMP: Bone morphogenetic protein
CD: Cluster of differentiation
CO₂: Carbon dioxide
EDTA: Ethylene diamine tetraacetic acid
ELISA: Enzyme-linked immunosorbent assay
FBS: Fetal bovine serum
HRP: Horseradish peroxidase
IL-1β: Interleukin-1β
ITS: Insulin-transferrin-selenium
MSC: Mesenchymal stem cell
NRS: Numeric rating scale
OA: Osteoarthritis
PBS: Phosphate buffered saline
PCR: Polymerase chain reaction
RA: Rheumatoid arthritis
TGF: Transforming growth factor
TKA: Total knee arthroplasty
TLR: Toll-like receptor
TNF-α: Tumor necrosis factor-α
